# Could vertigo be a post-COVID-19 sequela or presenting symptom?

**DOI:** 10.1186/s41983-023-00659-x

**Published:** 2023-05-11

**Authors:** Lamiaa I. Daker, Reham R. Elshafei, Mohammad Bahi, Asmaa Mohammed, Randa Erfan, Mohammed Gomaa

**Affiliations:** 1 Neurology Department, Faculty of Medicine, Fayoum University, Fayoum, Egypt; 2 Audio Vestibular Department, Faculty of Medicine, Fayoum University, Fayoum, Egypt; 3 Otolaryngology Department, Faculty of Medicine, Fayoum University, Fayoum, Egypt; 4 Medical Biochemistry and Molecular Biology Department, Faculty of Medicine, Fayoum University, Fayoum, Egypt; 5 Medical Biochemistry and Molecular Biology Department, Faculty of Medicine, Cairo University, Cairo, Egypt

**Keywords:** COVID-19, Vertigo, Video nystagmography

## Abstract

**Background:**

It has been postulated that COVID-19 has a substantial neuro-otological impact such as vertigo or dizziness that is rarely evaluated. The purpose of this research is to study the occurrence of vertigo (whether as presenting symptom or a sequela) and its etiological characteristics in patients with covid 19 infection and close contact**.** It is a cross-sectional study (convenient sample) conducted on patients that had a previous history of covid 19 infection and another group of contact individuals who presented with the sensation of vertigo**.** All the included participants underwent full neurological and otological examination, nasopharyngeal swab PCR to confirm COVID-19 infection and video nystgmograghy (VNG).

**Results:**

it was included 44 participants, where 7 (15.9%) of the participants were post-COVID-19 patients and 37 (84.1%) were close contacts of COVID patients. It was found that 6(85.7%) of post-COVID-19 patients had vestibular neuritis (VN), and 1(14.3%) patient had Benign Paroxysmal Positional Vertigo (BPPV). 9(23%) of those in close contact had positive PCR for COVID infection, 6(66.7%) of them had VN, and the other 3 (33.3%) had BPPV.

**Conclusion:**

Vertigo could be a possible complication or a presenting symptom in patients with COVID patients that is mainly attributed to peripheral vestibular dysfunction.

## Introduction

COVID-19 is a worldwide pandemic caused by SARS-CoV-2. Coronavirus belongs to Orthocoronavirinae, this subfamily is an RNA virus that could infect humans and birds [1]. Both the upper and lower respiratory tracts are susceptible to COVID-19 infection. The lung is most adversely affected by COVID-19, which gains access to host cells through the enzyme angiotensin-converting enzyme 2 (ACE2) [2].

It was hypothesized that SARS-CoV-2 possesses neurotropic and neuroinvasive behaviors like other coronaviridae, the virus has been found in the autopathy cerebrospinal fluid, but the precise process by which it enters the central nervous system (CNS) is still unknown. Given the low levels of ACE2 in the brain, it may first attack peripheral nerves [3]^.^ The virus may also enter the circulation from the lungs, penetrate the blood–brain barrier, and infiltrate the CNS, potentially inside an infected white blood cell [4].

Up to 30% of COVID-19 patients have been documented to experience neurological problems as a consequence. The inner ear is one of the neurological systems that the virus may attack, and the resulting damage may cause vertigo and/or tinnitus [5]. The goal of this study is to investigate the occurrence of vertigo as it may be a consequence of the COVID-19 infection or it may be the 1st presenting symptom of infection in close contact. So far, there is still a lack of scientific research on this topic; to our knowledge, this study is the first to address this issue in Egypt.

## Methods

This cross-sectional study (convenient sample) was carried out in the period from February to April 2022. The participants were divided into two groups: group (1) those who had previously gotten COVID-19 and had vertigo as a consequence of infection and group (2) of close contacts who just reported vertigo or dizziness. The contact group included in this study were outpatients who had recently complained of vertigo and sought medical advice. When interviewed, they revealed that they were close household contacts of COVID-19 patients—who were not participants in this study—so they were advised to perform nasopharyngeal PCR. Close contact was defined as a person who has had closer than < 6 feet for more than 15 min with people with a positive diagnosis of COVID-19, whether they were symptomatic or asymptomatic according to the Centers for Disease Control and Prevention (CDC) definition [6]. The eligible participants were assembled from the neurology and ENT outpatient clinics. Signed informed consent documents were provided by all the patients and the close contacts who participated in this study.

Patients with severe COVID-19 who required ICU admission were not included in this study. Patients who had previously undergone ear surgery and suffered head trauma, cardiovascular problems, or pregnant women that might have been a cause of vertigo, or who had a history of vertigo, dizziness, sensorineural hearing loss (SNHL), or were receiving ototoxic drugs such as chloroquine or hydroxychloroquine and other otological disorders before COVID-19 infection were also excluded from the study.

All the participants underwent a thorough neurological examination that included taking a thorough medical history of COVID-19 infection, presenting symptoms, duration of illness, medication received, associated comorbidities, and a neurological examination that focused on the analysis of vertigo complaints, including onset, course, duration, characteristics of the attacks, frequency, associated symptoms and triggering or alleviating factors.

The participants underwent the following battery of comprehensive audiological examinations including the Dix-Hallpike Positioning Test [7]: Turn the patient's head 45 degrees to one side and position them supine with their head over the edge of the examination bed, using both hands to support their head. If the patient has vertigo or nystagmus, conduct the test on the other side; the result is affirmative.

To diagnose benign paroxysmal positional vertigo (BPPV) it was required a history of brief episodes of vertigo induced by rolling the head from side to side while supine (positional vertigo) as well as a favorable result in the Dix Hallpike maneuverer [8]. The diagnosis of vestibular neuronitis was based on the rapid onset of severe rotatory vertigo, spontaneous horizontorotatory nystagmus, and an absence of neurologic symptoms that may reflect central nervous system involvement. [9].

The video nystagmography (VNG) evaluation was conducted with computerized two-channel monocular Micromedical Mobile Eyes, in which the camera may image the eye via a customized mirror in the audiology department. Oculography tests (smooth pursuit, saccade, and optokinetic), spontaneous nystagmus tests, gaze test, positioning tests, and caloric tests were VNG subtests and all were recorded. Pure tone audiometry and Acoustic immittance testing (Interacoustics AC40, and Interacoustics AT235 respectively, and both machines were calibrated according to the ISO standards) were done to exclude cases with chronic SNHL which might be a cause of vertigo.

Complete blood counts (CBC), erythrocyte sedimentation rates (ESR), C-reactive protein (CRP), serum ferritin, and liver function tests, which are significantly affected by COVID-19 infection.

The nasopharyngeal swabs of the included patients and close contacts who complained of vertigo were tested by a real-time fluorescence-based polymerase chain reaction (RT-PCR) to confirm the diagnosis of SARS-COV2 infection. The enrolled patients had a positive nasopharyngeal PCR, and particular symptoms that were indicative of COVID-19 infection, their most recent nasopharyngeal PCR was negative two weeks before participating in the study.

Computed Tomography (CT) of the chest was performed to determine if the patients had ground glass opacification indicative of associated pneumonia, using a CT scanner (Toshiba aquilion prime 160 slices, Japan) at the radiology department. On 1.5 Tesla magnetic resonance scanners (Toshiba Scanner Activion, Model TSX-031A 3D Volume, Japan).

### Statistical analysis

Data was gathered and coded to enable data manipulation before being double-entered into Microsoft Access and analyzed using the Statistical Package for Social Science (SPSS) software version 22 on Windows 7. (SPSS Inc., Chicago, IL, USA). Simple descriptive analysis of qualitative data in the form of numbers and percentages, arithmetic means as a measure of central tendency, and standard deviations as a measure of the dispersion of quantitative parametric data. The Independent samples t-test is used for quantitative data.

## Results

**Fig. 1:**
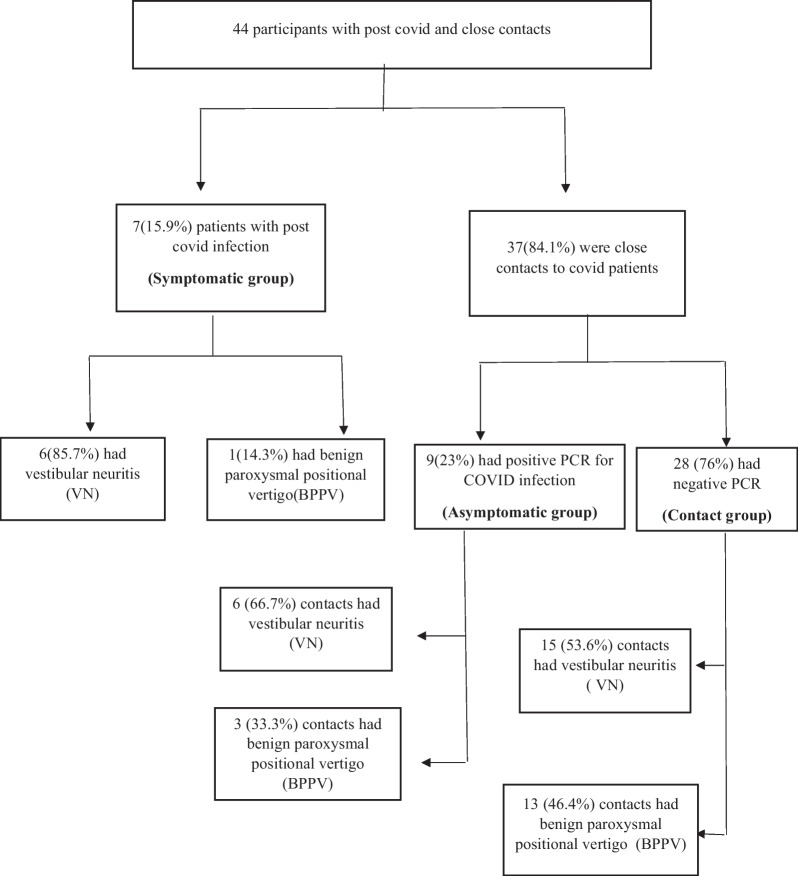
The distribution of the participants in this study

This study, it was included 44 participants, where 7 (15.9%) of the participants were post-COVID-19 patients (symptomatic group) and 37 (84.1%) were close contacts of COVID patients. It was found that 9 (23%) of them had a confirmed diagnosis of COVID-19 infection based on a positive nasopharyngeal swab(asymptomatic group) and 28 (76%)individuals had negative PCR results(contact group). The distribution of participant groups was illustrated in Fig.  1.

The age of the post-COVID-19 patients (symptomatic patients) ranged from 25 to 65 years with a mean of 49.8 ± 13.9 while it ranged from 31 to 81 with a mean of 52.2 ± 13 for the asymptomatic and contact groups. The demographic, clinical, and laboratory characteristics of the participants were shown in Table 1. None of the participants were vaccinated.

**Table 1:** The Demographic, clinical presentation, laboratory and neuroradiological data of the participants

	Post-Covid-19 patients (*n* = 7)	Close contacts (*n* = 37)
**Demographic data**		
Age (years) Mean ± SD	49.8 ± 13.9	52.2 ± 13
Sex (Male: Female)	1 (14.3%):6 (85.7%)	21(56.8%): 16(43.2%)
**Clinical presentation**		
Positional vertigo	1 (14.3%)	16 (43.2%)
Balance difficulties	6 (85.7%)	21 (56.8%)
**Laboratory data**		
**Serum ferritin**		
Normal	7 (100%)	33 (89.2%)
Elevated	–	4 (10.8%)
**CBC**		
Normal	7 (100%)	27 (73%)
Leucopenia	–	1 (2.7%)
Lymphopenia	–	6 (16.2%)
Lymphopenia and Leucopenia	–	1 (2.7%)
Lymphopenia and Leukocytosis	–	2 (5.4%)
**CRP**		
Negative	3 (42.9%)	23 (62.2%)
Positive	4 (57.1%)	14 (37.8%)
**Liver enzyme**s		
Normal	7 (100%)	33 (89.2%)
Elevated	–	4 (10.8%)
**CT chest at vertigo presentation**		
Normal	6 (85.7%)	36 (97.3%)
Fibrotic	1 (14.3%)	0 (0%)
GGO	0 (0%)	1 (2.7%)

*GGO* ground glass opacification

**Fig. 2:**
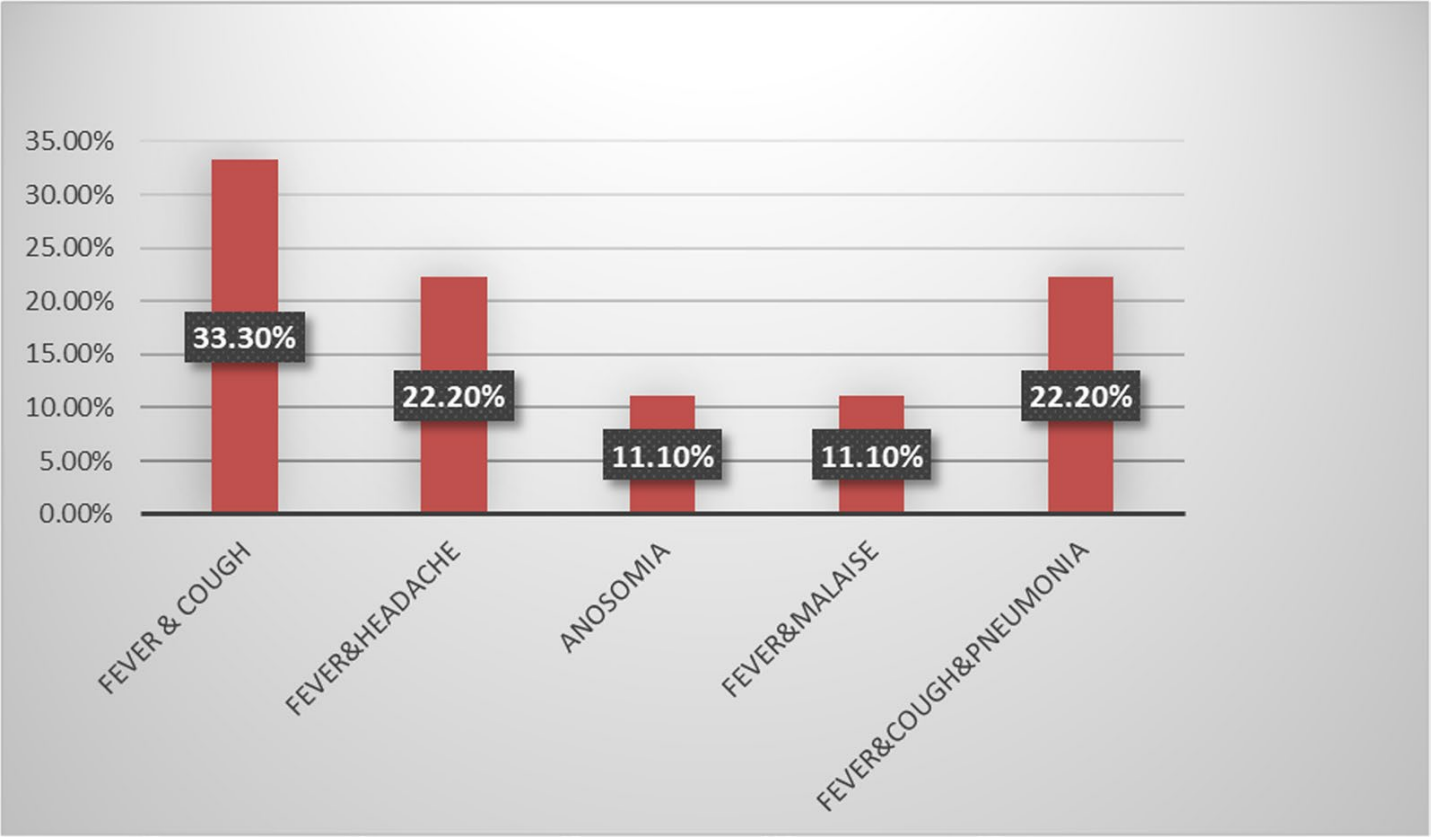
Clinical presentation of COVID-19 infection

As regards the post-COVID-19 patients, It was found that the duration of COVID infection ranged from 15 to 35 days with a mean of 22 ± 6.8, and the period that had elapsed since the patients had no symptoms and their nasopharyngeal swab PCR was negative was found to vary from 15 to 26 days with a mean of 21.1 ± 3.7. The mean duration from the onset of the COVID infection symptoms till the presentation of vertigo as a post-COVID sequela is 43.11 ± 8.6 days with a range of 30 to 57 days. Figure 2 illustrated the clinical manifestation of COVID-19 infection, and it was found that 1 (14.3%) patient required hospitalization while 6 (85.7%) got home management. At the time of COVID-19 illness, 4 (57.1%) patients had a normal CT chest, whereas 3 (42.9%) patients showed ground glass opacification. Table 1 illustrated the CT chest alterations at the time of the presentation of vertigo.

Regarding the close contact participants who had reported experiencing vertigo, the time elapsed from exposure to the infection to complaint of vertigo ranged from 3 to 11 days with a mean of 6.5 ± 1.93.

Concerning the video nystagmography (VNG) results, 6 (85.7%) of symptomatic patients had been diagnosed with vestibular neuritis (VN), 1 (14.3%) had benign paroxysmal positional vertigo (BPPV) affecting the posterior canal. In the asymptomatic and contact groups, it was discovered that 21 (56.8%) individuals had VN, 11 (52.4%) of them had right VN, and the remaining 10 (47.6%) had left VN. Additionally, 16 (43.2%) of the contacted individuals had BPPV, 12 (75%) of them had a posterior canal disorder, and the remaining 4 (25%) had a disorder of the horizontal canal as shown in Table 2.

**Table 2:** The video nystagmography (VNG) data of the participants

	Post-Covid-19 patients	Close contacts
	(*n*=7) (%)	(*n*=37) (%)
**Nystagmus**		
No	0 (0)	10 (27)
Gaze evoked	2 (28.6)	0 (0)
Positional	5 (71.4)	26 (70.3)
Mixed with Spontaneous	0 (0)	1 (2.7)
**Saccade**		
Normal	7 (100)	37 (100)
**Pursuit**		
Smooth	7 (100)	37 (100)
**Dix hall pike**		
Negative	6 (85.7)	21 (56.8)
Torsional nystagmus	1 (14.3)	12 (32.4)
Horizontal nystagmus	0 (0)	4 (10.8)
**OPK**		
Symmetric	7 (100)	37 (100)
Not symmetric	0 (0)	0 (0)
**Caloric test**		
Negative test	1 (14.3)	16 (43.2)
Right canal weakness	2 (28.6%)	11 (29.7)
Left canal weakness	4 (57.1)	10 (27)
**Final diagnosis**		
**BPPV**	1 (14.3)	16 (43.2)
Posterior canal	1 (100)	12 (75)
Horizontal canal	0 (0)	4 (25)
**Vestibular neuritis**	6 (85.7)	21 (56.8)
Right	2 (33.3)	11 (52.4)
Left	4 (66.7)	10 (47.6)

*OPK* Optokinetic, *BPPV* Benign Paroxysmal Positional Vertigo

It was shown that in the asymptomatic group with positive PCR, it was discovered that 3 (33.3%) individuals had BPPV and 6 (66.7%) individuals had VN, whereas, in the contact group with a negative COVID PCR, 13 (46.4%) individuals had BPPV and 15 (53.6%) contact persons had VN.

## Discussion

The coronavirus pandemic that began in 2019 has offered a variety of challenges for both patients and clinicians. The ongoing SARS-COV-2 epidemic is thought to have resulted in millions of severe cases and deaths worldwide. The condition is primarily defined by an acute respiratory problem, but it also produces various neurological symptoms such as smell and taste issues, headache, and confusion. Post-covid neurological complications have been documented during and after recovery [10].

Few studies linked vestibular dysfunction with COVID-19 are reported in the literature. Such papers mainly discuss case reports or only investigate symptoms [11]. To our knowledge, it is the first research that studies the association between SARSCOV-2 and vertigo whether vertigo could be a consequence of COVID-19 infection or it might be 1^st^ presenting symptom, and discusses its etiological characteristics in Egyptian patients. In this study, it was found that participants that had a previous history of COVID-19 infection and reported vertigo as a post-COVID sequela were middle age with a mean of 49.8 years and showed female predominance (85.7%) similar to [12] that postulated prevalence of post-COVID sequela is Common in middle age and increased with age, with a 3.5% rise every decade of life regardless of the severity of COVID symptoms.

Many COVID-19 survivors do not fully recover, presenting new or persistent symptoms lasting weeks or even months, regardless of the severity of the initial infection [13]. The NICE guidelines on COVID-19,2022 [14] stated a definition for post-COVID-19 syndrome distinguishing it from the acute phase of the infection with symptoms lasting up to 4 weeks or developing new symptoms between weeks 4 and 12 after infection as replication-competent SARS-CoV-2 has not been isolated after 3 weeks. In this study, it was shown that the mean duration from the onset of the infection till the presentation of vertigo is 43.11 ± 8.6 days. The mechanisms underlying the post-COVID syndrome are poorly understood. Yet, it was thought to be multifactorial including exaggerated inflammatory response, neurotransmission alterations, persistent viral damage, and functional alterations [15].

It has been suggested that vertigo is not a common symptom of post-COVID-19 syndrome. The survivors usually reported a post-viral syndrome of chronic malaise, diffuse myalgia, and depressive symptoms. Gallus and colleagues,2021 [16] investigated 48 patients with a history of mild COVID-19 symptoms and reported that 8.3% had dizziness, and 8% had balance difficulties one month after COVID infection.

Previous clinical reports and case studies had suggested audiovestibular dysfunction could be a possible post-COVID complication, particularly in non-hospitalized patients [17–19]. According to these data, the most prevalent mechanisms of vestibular neuritis in post-COVID patients were a direct viral invasion of the vestibular nerve or a post-viral inflammatory response. The detection of T4 T-helper and T8 T-suppressor cells by specific monoclonal antibodies in inner ear illnesses supported the postviral immunological theory [20]. Furthermore, since the inner ear is particularly susceptible to ischemia, vascular involvement may be one of the potential complications of COVID-19, along with COVID-19-induced hypercoagulability and microthrombi formation [21]. The previously described mechanisms may provide a reasonable explanation for our clinical and VNG data results since vestibular neuritis was shown to account for 85.7% of post-COVID patients, which is consistent with prior clinical studies [17–19].

Furthermore, it was shown only one patient (14.3%) experienced BPPV with posterior canal preference, and he was hospitalized during the COVID-19 infection. The prolonged immobilization and hospitalization of patients during COVID infection may increase the incidence of BPPV resulting in otolith detachment, with the posterior canal being severely impacted due to extended pronation to maintain adequate oxygenation [8]. The majority of the post-COVID-19 participants (85.7%) in this study were not hospitalized and the participants had a relatively short mean duration of illness might be responsible for the low proportion of BPPV in our study.

It was postulated that vertigo could be the initial and sole presenting symptom of COVID-19 infection, as it was shown that 23% of those who were in close contact with COVID patients (asymptomatic group) experienced vertigo. Saniasiaya and Kulasegarah [22] analyzed 14 different studies that included data from 141 coronavirus patients and they found that dizziness was the initial symptom in three (2.13%) of the patients and, in two of them, it was followed by respiratory symptoms. Currently, neither the Centers for Disease Control and Prevention (CDC) nor the World Health Organization (WHO) listed dizziness or vertigo as a symptom of COVID-19 [23].

So far, published case reports or studies have only mentioned dizziness as a presenting symptom of COVID infection. Neither of these studies carefully examine patients with vertigo by VNG or radiological investigation to identify what the most likely causes are beyond this symptom, whether it was merely dizzy spells or suggested a possible peripheral or central virus invasion.

It was found that 66.7% of the asymptomatic group reported abnormal VNG data indicating VN, and 33.3% had positional nystagmus followed by caloric weakening suggestive of BPPV. Baig and colleagues [3], suggested that the virus invades neural tissue via circulation and binds to angiotensin-converting enzyme 2 receptors located in the capillary endothelium and early direct nerve invasions such as cytomegalovirus, rubella, and adenoviruses.

The contact group with negative PCR revealed that 46.4% of participants had BPPV and 53.6% had VN. People's behavior has changed as a result of the pandemic's isolation. A lack of social interaction leads to the onset and/or worsening of depression and anxiety, as well as a decline in sleep quality, sedentary lifestyles, and dietary errors. Abuse of sweets, which causes weight gain, may advocate a state of low immunity, which may increase the incidence of other viral infections resulting in VN. The practice of working from home, frequently with insufficient facilities, leads to postural unhealthy habits that cause cervical pain or may provoke BPPV [24].

## Conclusion

We would like to emphasize that vertigo should be taken seriously as it has been proven to be a significant clinical manifestation whether it is a presenting symptom or possible sequela among COVID-19 patients.

## Recommendations

Persistent vertigo with COVID-19 infection warrants referral to the Neurology and Otorhinolaryngology Departments for thorough examination and investigation. Furthermore, for stable COVID-19 patients, we recommend vestibular rehabilitation therapy, which has shown promising results.

## Limitations

This study had some limitations including a small number of participants and a lack of study on the effect of COVID vaccination on experiencing vertigo.

## Data Availability

The dataset cannot be publicly available due to institutional rules. The datasets used and/or analyzed during the current study are available from the corresponding author upon reasonable request.

## Abbreviations

ACE: Angiotensin-converting enzyme
BPPV: Benign paroxysmal positional vertigo
CBC: Complete blood counts
CNS: Central nervous system
COVID-19: Coronavirus disease 2019
CRP: C-reactive protein
CT: Computed tomography
ESR: Erythrocyte sedimentation rates
SARS-CoV-2: Severe acute respiratory syndrome coronavirus 2
SNHL: Sensorineural hearing loss
VN: Vestibular neuritis
VNG: Video nystagmography
